# Who Needs Bereavement Support? A Population Based Survey of Bereavement Risk and Support Need

**DOI:** 10.1371/journal.pone.0121101

**Published:** 2015-03-26

**Authors:** Samar M. Aoun, Lauren J. Breen, Denise A. Howting, Bruce Rumbold, Beverley McNamara, Desley Hegney

**Affiliations:** 1 School of Nursing and Midwifery, Faculty of Health Sciences, Curtin University, Perth, Western Australia, Australia; 2 School of Psychology and Speech Pathology, Faculty of Health Sciences, Curtin University, Perth, Western Australia, Australia; 3 Palliative Care Unit, Department of Public Health, La Trobe University, Melbourne, Victoria, Australia; 4 School of Occupational Therapy and Social Work, Curtin University, Perth, Western Australia, Australia; 5 Centre for Nursing Research, Sir Charles Gairdner Hospital, Perth, Western Australia, Australia; 6 School of Nursing and Midwifery, University of Southern Queensland, Brisbane, Queensland, Australia; The George Institute for Global Health, INDIA

## Abstract

This study identifies and describes the profiles of bereavement risk and support needs of a community sample in Australia and tests the fit of the data with the three-tiered public health model for bereavement support. Family members who were bereaved 6–24 months prior to the survey and who were clients of four funeral providers participated (May-July 2013). A postal survey was used to collect information about bereaved people’s experience of caring and perceived satisfaction with any bereavement support provided. The questionnaire included a validated risk assessment screening measure for Prolonged Grief Disorder (PG-13). A total of 678 bereaved people responded. The model predicted that 60% of the sample would be low risk, 30% moderate risk, and 10% high risk. Actual figures were very close at 58.4%, 35.2% and 6.4% respectively. The analysis of the demographic characteristics, experience and impact of caring and bereavement, and satisfaction with support received from a variety of sources revealed differential experiences and needs that align with the expectation of low, moderate, and high bereavement support need, as articulated in the public health model. This is the first empirical test of the public health model of bereavement support. As there is a lack of clear evidence to guide development and allocation of bereavement support programs, the findings have the potential to inform the ability of services, community organizations and informal networks to prioritize care according to each level of bereavement need. This is essential to achieve cost-effective and equitable resource allocation.

## Introduction

The death of a loved one is often a significant life stressor with effects across emotional, physical, behavioral, cognitive, social, spiritual and financial domains. While grief responses following bereavement are unique to each individual, there is an increasing recognition that bereavement can precipitate ongoing psychiatric distress in some people [1]. The evidence distinguishing normal and pathological grief reactions, typically called Prolonged Grief Disorder (PGD) [2] or Complicated Grief [3], is mounting. The latest edition of the Diagnostic and Statistical Manual of Mental Disorders (DSM-5) removed bereavement as an exclusion criterion for both Major Depressive Disorder and Adjustment Disorder and included Persistent Complex Bereavement Disorder as a condition for further study [4]. Furthermore, the forthcoming International Classification of Diseases (ICD-11) proposed the inclusion of PGD as a new classification [5]. The distinction between normal and prolonged grief is also supported by surveys of psychologists [6] and the wider community [7].

Current estimates suggest that 10 to 20% of bereaved individuals demonstrate persistent psychiatric difficulties [2,8]. People experiencing these psychiatric difficulties as a result of bereavement are most likely to benefit from targeted grief interventions [9–11]. By contrast such interventions typically demonstrate limited to no benefit for the majority of grievers and may even be detrimental [12]. However, it is concerning that people with PGD are less likely to seek professional help [13]. It is important to note that these prevalence estimates are derived from non-representative samples of bereaved people accessed via hospital records [14], psychiatric clinics [15], and community services for retirees and widows [16]. There are no accurate data on the population prevalence of PGD so there is a clear need to determine the population prevalence of PGD.

The most comprehensive strategy for bereavement support in many communities is offered by palliative care services, which provide care to patients with terminal illnesses and to their family caregivers before and after the patient’s death. Formal policies and standards of care propose that supports should be offered according to need [17–22]. Despite these policies, surveys of palliative care services demonstrate that the offer of supports and services to bereaved families may have limited congruence with assessments of support need [23–25]. Approximately two-thirds of Australian palliative care services report using some form of bereavement risk assessment with family caregivers, usually prior to the death. However, the utility of these assessments varies widely and often rests upon the subjective opinion of staff members or the use of a non-validated screening tool [26]. The situation is complicated by the lack of clear evidence to guide the development and allocation of cost-effective bereavement support services [26–28]. Providing universal bereavement support irrespective of need is neither effective nor economical [27,29].

One conceptual framework which has recently been developed to guide bereavement risk assessment is the public health model of bereavement support [29–31], which comprises a three-tiered approach to bereavement risk and need for support wherein the low risk group (first tier) would need support principally from family and friends, the moderate risk group (second tier) would need support from the wider community through some general support from various professionals, and the high risk group (third tier) would need support from mental health services. The operationalization of this model would provide evidence to guide the development and allocation of cost-effective bereavement support services, which is identified as an important gap in the literature [26–28]. Thus, the identification of the prevalence of bereavement risk will inform policy and practice in the provision of support and services following bereavement.

To guide the development of evidence-based interventions, an understanding of the bereavement risk and support needs of bereaved people is essential, and may be uncovered via a population-based survey of bereaved people. Such surveys are called mortality follow-back surveys with bereaved relatives and are common in many countries [32–34].

The objectives of this article are to determine through a survey the proportion of bereaved people in a general population sample who meet the criteria for PGD; to outline the profile of the different risk groups; to identify the sources of bereavement support people accessed and their perceived unmet needs for support, according to bereavement risk; and to test the fit of the data with the public health model of bereavement support.

## Methods

Ethics approval was granted by the Curtin University Human Research Ethics Committee (HR-57/2012).

### Study design

The study is a population-based cross-sectional investigation of bereavement experiences. A postal survey was used to collect information from clients of four funeral providers in Australia, specifically from metropolitan and regional areas of Western Australia and Victoria (May-July 2013), 6 to 24 months after the death of their family member. We chose this time period as 6 months post-bereavement is the earliest time period required for diagnosis of PGD while 24 months is not likely to compromise the accuracy of recalled information. Funeral providers were engaged as it was not possible to recruit through the Death Registry.

### Participants and Procedure

A total of 3,190 study packages were delivered to the four funeral providers. These packages contained an invitation letter addressed from the funeral provider to the family, information sheet, the questionnaire, a list of support services for the family to use in case the respondent became distressed while completing the questionnaire, and a reply paid envelope. The funeral providers then selected clients who were bereaved 6–24 months ago from their databases, attached names and address labels on the envelopes and mailed the study packages. Consent was implied by the return of the completed survey. No reminder letter was sent as it was felt to be too intrusive on the bereaved families. Clients were eligible to participate in the study if they had been bereaved by a close family member or friend in the specified timeframe, were able to read, understand and write in English, and were over 18 years of age.

### Materials

A questionnaire was developed to obtain demographic information; the supports people accessed; supports they would have liked to have been able to access; their perceived needs and whether they were met. It has eight sections with a total of 82, predominantly closed, questions. The questionnaire was developed in consultation with a reference group comprising representatives of the funeral industry, bereavement counselors, palliative care services, primary care, and community based services. The survey was pilot tested and questions were found acceptable and feasible [30].

The questionnaire includes a validated risk assessment screening measure for PGD, the PG-13 [2]. Compared to other tools reviewed, the PG-13 is short, easy to self-administer, has a theoretical basis and aligns with the criteria proposed for inclusion in the forthcoming World Health Organization’s International Classification of Diseases (ICD-11) [2,5]. The PG-13 measures responses to separation social/functional impairment, and cognitive, emotional and behavioral symptoms over a period of not less than 6 months since bereavement. All 5 criteria must be met to indicate the presence of PGD: event (bereavement); separation distress; duration (i.e., >6 months); cognitive, emotional and behavioral symptoms; and social/occupational impairment. The score range is 11–55 and a score of 36 or more is a clinical indicator of PGD.

### Analysis

Descriptive statistics for variables were calculated: frequencies and proportions for categorical variables; means, standard deviations, medians, minimums and maximums for continuous/discrete variables. The PG-13 responses were scored according to the developers’ instructions [2,35]. Significance testing was performed using chi-square for categorical variables, and non-parametric tests for the median for the non-Normally distributed continuous variables. Significance was set at the p = 0.05 level for the study. Analysis was carried out using IBM SPSS Statistics Version 22. The open ended responses were manually coded using an open content analysis process [36].

## Results

Six hundred and seventy eight questionnaires were returned completed. The response rate was 21.3%.

### Demographic Characteristics of Respondents

Seventy one percent of the bereaved people who responded were female; mean age of 62.4 years (SD 12.2); 49.6% were married and 37.0% widowed; 36.8% were the spouse of the deceased, and 45.3% were the son or daughter of the deceased; 70.1% were Australian, 45.2% had finished high school, 30.5% had a diploma or trade qualification and 19.6% had a university degree; 36.9% were currently employed, and 38.9% were retired (Table 1). The mean period of bereavement for respondents was 14.3 months (SD 6.3). The mean age of the deceased was 75.4 years (SD = 18.3).

**Table 1:** Demographic characteristics of the bereaved.

	Total, n = 678	
	n	%
**Gender**		
Male	194	28.8
Female	479	71.2
**Age** (years)		
Mean (SD)	62.4	(12.2)
Median(Range)	62.00	(20–96)
**Marital status**		
Single/never married	25	3.7
Married/de facto	336	49.6
Separated/divorced	59	8.7
Widowed	251	37.0
**Cultural background**		
Australian	475	70.1
Other English Speaking background	125	18.4
Non English Speaking background	37	5.5
**Highest level of education**		
No formal education	3	0.4
Primary school	28	4.2
High school	304	45.2
Diploma/ certificate/ trade qualification	205	30.5
University degree	132	19.6
**Employment**		
Paid employment	247	36.9
Retired	260	38.9
Disabled	9	1.3
Household duties	92	13.8
Unemployed	15	2.2
Other	46	6.8
**Relationship to the deceased**		
Spouse/partner	249	36.8
Parent	48	7.1
Sibling	25	3.7
Daughter/son	307	45.3
Other relative	32	4.7
Friend	13	1.9
Other	3	0.4
**Period of Bereavement**: Mean (SD) in months	14.3	(6.3)

### The three risk groups

The 580 respondents who completed the PG-13 were grouped into three categories of risk based on the predominance and severity of the diagnostic criteria (Table 2):

1. Those with high level of risk (meeting the five criteria for PGD): 6.4% (n = 37) with median equals 48 and scores ranging between 39 and 55 (mean = 47, SD = 4.5);
2. Those with moderate level of risk (meeting three or four PGD criteria): 35.2% (n = 204) with median equals 27, and scores ranging between 13 and 54 (mean = 27.7 SD = 7.9); and
3. Those with low level of risk (meeting one or two PGD criteria): 58.4% (n = 339) with median equals 15 and scores ranging between 11 and 32 (mean = 16.2 SD = 4.8).

**Table 2:** PGD criteria and PG-13 scores for the 3 groups (p-values <0.001).

	Low risk (1–2 criteria)		Moderate risk (3–4 criteria)		High risk (5 criteria)	
**PGD criteria met:**	n	%	n	%	n	%
Event criterion	339	100.0	204	100.0	37	100.0
Separation distress	39	11.5	195	95.6	37	100.0
Duration criterion	40	11.8	190	93.1	37	100.0
Social & Functional Impairment	22	6.5	53	26.0	37	100.0
Cognitive, Emotional & Behavioral Symptoms	0	0	25	12.3	37	100.0
**PG-13 score:**						
Median (Range)	15	(11–32)	27	(13–54)	48	(39–55)
Mean ± SD	16.2	±4.8	27.7	±7.9	47.0	±4.5

Pearson’s Chi-Square Test for categorical variables, Independent samples Kruskal-Wallis Test for continuous variable.

All bereaved people in the high risk group reported meeting all 5 criteria of PGD (100%). In the moderate risk group, the majority exhibited separation distress (95.6%) and the duration criteria (93.1%) and to a lesser, but considerable extent, social and functional impairment (26%), and to a much lesser extent, cognitive emotional or behavioral symptoms (12.3%). The low risk group did not exhibit any cognitive emotional or behavioral symptoms; less than 12% exhibited separation distress and the duration criteria and less than 7% reported social and functional impairment (Table 2).

A two-way chi-square revealed large, statistically significant differences in observed frequencies for each of the PG-13 criteria between the three risk groups. Additionally, a Kruskal-Wallis test indicated statistically significant differences in the median risk scores between risk groups (p<0.001). Post-hoc analysis of pairwise comparisons between the PG-13 scores for low risk and moderate risk, low risk and high risk, moderate risk and high risk, were all statistically significant (p = <0.001) (Table 2).

### Sources and perceived usefulness of bereavement support

**Fig 1:**
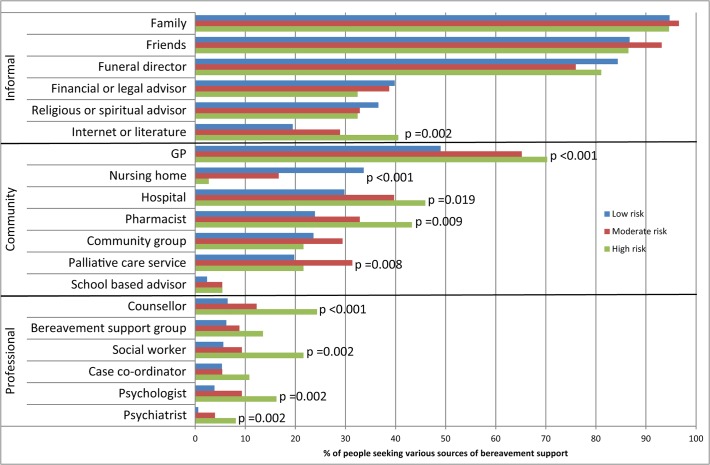
Sources of bereavement support accessed grouped according to types of support*. *Only categories with statistically significant differences (p<0.05) are displayed on chart (Pearson’s Chi-Square Test).

The majority of the bereaved respondents in each of the 3 risk groups accessed support from family and friends, followed by funeral directors and GPs (Fig. 1). Access to mental health professional sources of support (counsellor, social worker, psychologist and psychiatrist) was more frequently reported by the high risk group. The moderate risk group was particularly visible in the support accessed from community groups and palliative care services. There was a statistically significant difference in observed frequencies of sources of support between the three risk groups, p-values ranging from 0.019 to <0.001 (Fig. 1).

The higher the risk group, the more likely there was perceived lack of support (Table 3). While 71% of those in the low risk group felt they had enough support and 17% did not, only a quarter of the high risk group felt they had enough support and almost two-thirds did not. Differences in perceived support between the three groups were statistically significant (p<0.001).

**Table 3:** Perceived bereavement support by risk groups (p-value<0.001).

Bereavement support	Low risk		Moderate risk		High risk	
	n	%	n	%	n	%
Enough support	231	70.6	119	60.7	9	25.0
Not enough support	64	19.6	60	30.6	23	63.9
Did not need support	26	8.0	10	5.1	1	2.8
Other	6	1.8	7	3.6	3	8.3

Pearson’s Chi-Square Test.

### Bereavement risk profiles

Analyses revealed several significant differences between the characteristics of the three groups of bereaved people (Table 4). In terms of the characteristics of the bereaved people themselves, sex, age, marital status, education, employment and relationship to deceased all differed significantly between the three groups. There were also several differences regarding characteristics of the deceased in terms of sex, age, place of death and cause of death. Finally, the impact of the death on the bereaved individual’s health also differed significantly between the three risk groups. The following three profiles reflect these specific differences.

**Table 4:** Profile of bereaved people by risk groups.

Variable N = 580	Low risk (1–2 criteria PG-13) n = 339		Moderate risk (3–4 criteria PG-13) n = 204		High risk (5 criteria PG-13) n = 37		*p*-value
	n	%	n	%	n	%	
***Characteristics of the Bereaved***							
**Sex**							*0*.*002*
Male	115	34.1	50	24.5	4	11.1	
Female	222	65.9	154	75.5	32	88.9	
**Age** (years)							
Mean (±SD)	61.1	(±11.2)	63.4	(±13.2)	55.7	(±11.8)	*0*.*001*
Median (range)	62.0	(20–89)	63.0	(27–93)	57.0	(30–79)	
**Marital status**							*<0*.*001*
Never married/single	14	4.2	5	2.5	4	11.1	
Married/de facto	211	62.8	69	34.0	12	33.3	
Separated/divorced	34	10.1	13	6.4	3	8.3	
Widowed	77	22.9	116	57.1	17	47.2	
**Education**							*0*.*008*
Primary school	6	1.8	15	7.4	2	5.6	
High school	141	42.0	97	47.5	20	55.6	
Diploma/cert.	106	31.5	60	29.4	9	25.0	
University	83	24.7	32	15.7	5	13.9	
**Employment**							*0*.*003*
Paid employment	139	41.5	68	33.5	13	36.1	
Retired/volunteer	127	37.9	79	38.9	6	16.7	
Disabled	6	1.8	1	0.5	0		
Home duties	36	10.7	35	17.2	7	19.4	
Unemployed	7	2.1	5	2.5	3	8.3	
Other	20	6.0	15	7.4	7	19.4	
**Relationship to deceased**							*<0*.*001*
Spouse/partner	74	21.8	116	56.9	17	45.9	
Parent	9	2.7	23	11.3	11	29.7	
Sibling	15	4.4	6	2.9	2	5.4	
Offspring	206	60.8	52	25.5	5	13.5	
Other relative	24	7.1	4	2.0	1	2.7	
Friend	8	2.4	3	1.5	1	2.7	
Other	3	0.9	0		0		
***Characteristics of the deceased***							
**Age**							*<0*.*001*
Mean (±SD)	80.6	(±14.7)	69.04	(±19.4)	52.67	(±23.1)	
Median (range)	84.0	(0–103)	74.00	(0–97)	53.50	(0–93)	
**Sex**							*<0*.*001*
Male	148	44.2	119	60.1	25	69.4	
Female	187	55.8	79	39.9	11	30.6	
**Place of death**							*<0*.*001*
Home	34	13.1	31	21.4	8	44.4	
Hospital	95	36.5	66	45.5	7	38.9	
Hospice	22	8.5	18	12.4	1	5.6	
Nursing home	106	40.8	24	16.6	1	5.6	
Other	3	1.2	6	4.1	1	5.6	
**Cause of death**							*<0*.*001*
Life limiting illness	253	75.3	120	60.3	13	36.1	
Non-life limiting illness	83	24.7	79	39.7	23	63.9	
***Impact on the health of the bereaved since death***							
**Physical health**							*<0*.*001*
Improved	49	14.5	14	6.9	1	2.7	
Stayed the same	246	72.6	115	56.9	10	27.0	
Got a bit worse	39	11.5	63	31.2	18	48.6	
Got a lot worse	5	1.5	10	5.0	8	21.6	
**Mental health**							*<0*.*001*
Improved	55	16.3	15	7.4	1	2.7	
Stayed the same	239	70.7	105	52.0	10	27.0	
Got a bit worse	41	12.1	68	33.7	9	24.3	
Got a lot worse	3	0.9	14	6.9	17	45.9	
**Financial situation**							*<0*.*001*
Improved	89	26.3	27	13.3	3	8.1	
Stayed the same	212	62.5	123	60.6	16	43.2	
Got a bit worse	33	9.7	40	19.7	11	29.7	
Got a lot worse	5	1.5	13	6.4	7	18.9	

#### Low Risk: Grieving for a parent

Typically the bereaved person in this group is about 60 years of age, usually married, and compared to the higher two risk groups, the deceased is much older (about 80 years) and usually a parent. Death mostly occurred in nursing homes or hospital and 75% of deaths were due to terminal illness or old age/dementia. The majority of the bereaved people in the low risk group perceived they got enough support from the services caring for their relative, and did not need more. Their physical and mental health was not greatly affected by the death. The following quote is typical of this low risk profile:

*Mum was ready to leave this world*, *she said she’d had a long and happy life and now was the time to leave*. *I was most grateful that she really didn’t have a lot of suffering before she passed*. *Most of her children were at the bedside when she passed and the feeling was quite peaceful in the room*. *(ID 1244)*

#### Moderate Risk: Grieving for a spouse

In this group, the bereaved person is about 63 years of age and usually widowed. The age of the deceased tends to be closer to the age of the bereaved person (69 years) and the deceased is usually a spouse. Most deaths occurred in hospital, with similar rates of cause of death due to non-life limiting illnesses (39%) and life limiting illnesses (36%) in this profile. Feelings of their physical and mental health getting a bit worse were more pronounced than with the lower risk group. The following quotes from three respondents are typical of this moderate risk profile:

*Time does not heal*, *just brings acceptance and resignation*, *acceptance of God’s will*, *resignation to loss*. *(ID 1621)*

*It was painful and heartbreaking to watch him lingering and dying*, *no quality of life*, *but it was a relief when he passed away*. *I felt deeply comforted and at peace that I was by his side when he took his last breath (ID 1270)*

*After my husband died I knew I had to carry on so did not dwell on feeling sorry for myself*. *Although at times it is lonely but I have a lot of interests*, *church…*. *(ID 1248)*

#### High Risk: Grieving for a spouse (of a younger age) or a child

Compared to the two lower risk groups, both the bereaved person (55yrs), and the deceased (52 years) in the high risk group are typically younger. This bereaved group has a high proportion of spouses (46%) and parents (30%). Death occurred mainly at home or hospital and 64% of deaths were due to non-life limiting illnesses. Feelings of their physical and mental health getting a lot worse were more pronounced than for the two lower risk groups. Nearly two-thirds did not get enough support, and the majority needed more professional support, community based support and information. The following quote is typical of this high risk profile:

*I have a new respect for Mental Health*. *I am still suffering on a daily basis*. *I'm not a happy person*. *I'm not smiling*. *I'm not cooking for myself*. *I don't go out*. *I don't want to go to bed at night due to the quietness*. *I cannot sleep on the other side of the bed*. *I cry several times a day*, *I cry at night*. *Some days I can hardly function—I spent 4 days in bed 2 weeks ago*. *My immediate family are not equipped to identify but know about my depression—there is a distance in between my siblings and my children's homes (kms)*. *I feel like I might die alone*. *My mother calls me every other day*. *Doesn't know what she can do*. *(ID 1216)*

## Discussion

**Fig 2:**
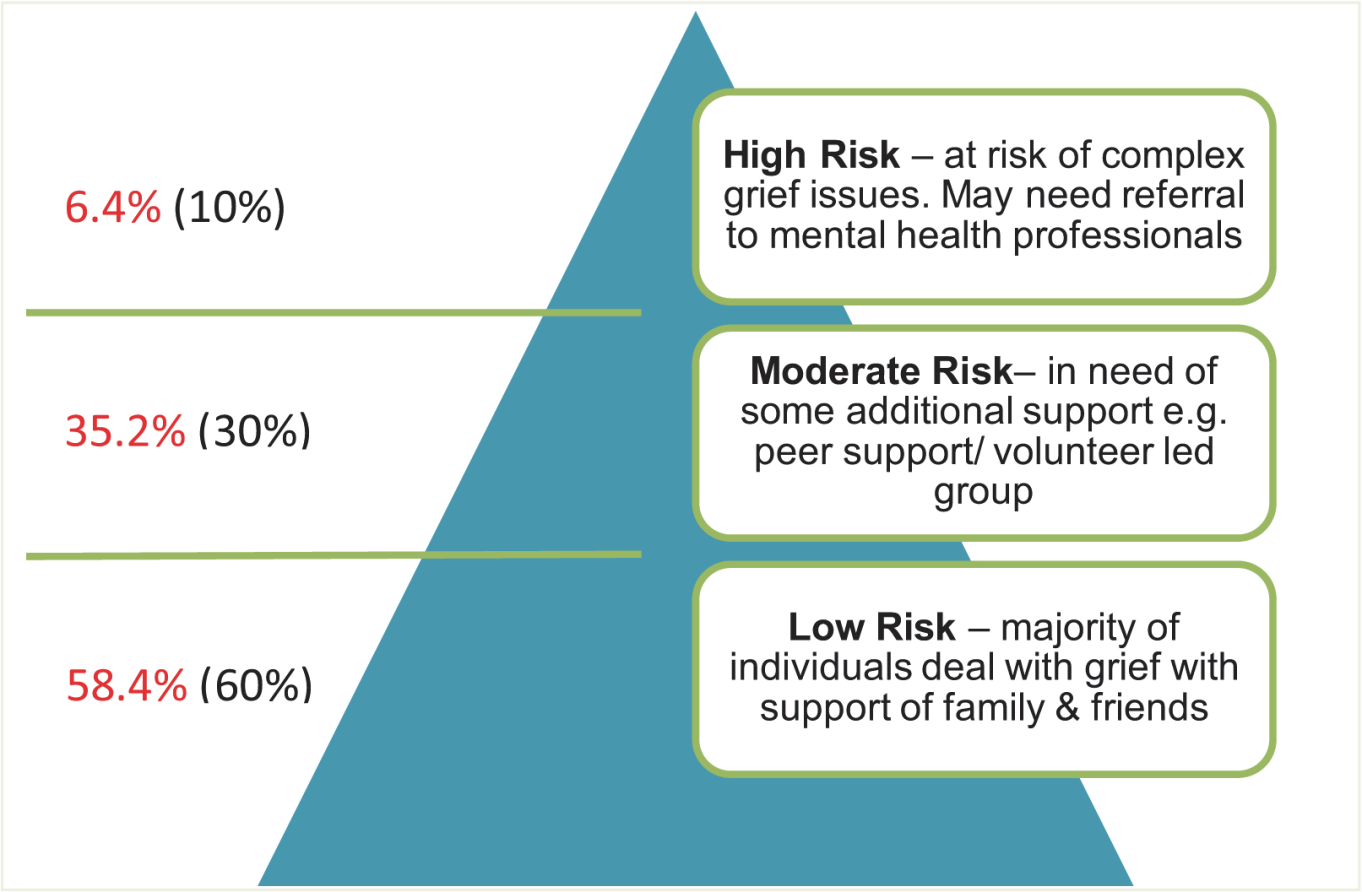
The Public Health Model: Predicted (in brackets) and Actual Proportions for the three risk groups.

The analysis of the demographic characteristics, type of bereavement support, and perceived support received from a variety of sources revealed differential experiences and needs that align with the expectation of low, moderate, and high bereavement risk, as articulated in the public health model (Fig. 2). The proportion of the bereaved in each risk level obtained from this analysis come very close to the proportions we proposed in the theoretical framework of the public health model [29,30]: The predicted and actual proportions of low risk were respectively 60% vs 58.4%; for moderate risk 30% vs 35.2%, and for high risk 10% vs 6.4%.

The survey was constructed to provide information on the population-based experiences of bereavement, including the extent of the alignment of bereavement risk and service need. The validated PG-13 means that we were able to determine a population rate of PGD in this survey of 6.4%. In addition, the low and moderate risk groups can be distinguished clearly on the basis of PG-13 scores as well as the sources of support (mental health professional sources for high risk, community support for moderate risk and family and friends for low risk). Additionally, the profiles provide a context for the scores: low scores typically arose from losses that were expected and less disruptive to daily routines (e.g., the death of an aged parent); moderate scores typically arose from deaths that were both expected and everyday-disruptive (e.g., the death of an ageing and ill partner); and deaths that are both unexpected or resisted and disruptive (such as a child or a younger spouse) lead to high scores on the PG-13.

Further, in addition to revealing that there might be a difference in type of response between the three groups, the data also show differences in degrees of support need across the three groups. The sources of support listed by respondents indicate that for the most part those in the low risk group were satisfied with support provided through everyday interactions. Those in the moderate risk group were also satisfied with the level of support, but most of them were also linked with some sort of bereavement follow-up program, through palliative care or a community support group. Those in the high risk group considered the support they received to be inadequate; such needs necessitate targeted mental health interventions in addition to other forms of support that were effective for people in the lower risk groups.

It is worth noting that the moderate risk group (second tier in Fig. 2) was identified in our consultations as causing challenges for palliative care services, as they sought to provide timely and adequate support so bereaved people did not end up in the third tier with prolonged grief [37]. This middle tiered proportion is clearly reflected in the UK Sobell House’s analysis of retrospective data on risk assessment and type of support provided for bereaved family caregivers (1989–2002) [38]. As such the support for this tier would be provided by non-specialist social and therapeutic support such as volunteer bereavement workers, bereavement mutual-help groups, and faith-based and other community groups.

The high-risk group involves predominantly ‘out of time deaths’. The supposition that PGD arises in people who lack adequate support seems unfounded: the high-risk group here accesses the same informal and community support as the other groups; but for them it is not enough. Not only do members of this group access bereavement specific mental health professional support, there is a hint that they may actively seek this out in their greater use of internet and educational resources. This differs from previous findings that people with higher risk for PGD were less likely to seek support [13].

The high level of reported support from funeral directors deserves further investigation. Most sources of support are found in relationships that were already in place during the person’s extended dying time; but funeral directors might be expected to have quite a brief involvement in comparison with most of these other roles. It is unclear whether this means that funeral directors are providing ongoing support, or whether the quality of their interaction in the immediate post-death period has a lasting positive effect. It is also worth noting that some of the reported sources of bereavement support appear to be non-specific; that is, it comes from people who are simply doing their jobs well, including some who might not normally be thought of as providing bereavement support (for example, financial and legal advisors).

The findings indicate that support needs of bereaved people at different levels may be met using different combinations of strategies. Rather than a ‘one-size-fits-all’ approach to bereavement care, there is merit in providing flexible and targeted bereavement services, many of them informal and within local communities as well as formal offerings of health services [31].

### Limitations

In the absence of access to identifiable data from death registries in Australia (whereas these data sources are accessible in the UK), databases of funeral providers can be an alternative source to reach bereaved people. Engaging funeral providers as research partners in this project is a first nationally and internationally. The response rate is low but comparable to what is expected from anonymous population based postal surveys of bereaved people [33,39]. Typically, service based surveys would have higher response rates than population based surveys, as respondents would have been engaged with the service prior to receiving the survey [34]. Nevertheless, our respondents’ profile is comparable with the profile of a recent mortality followback survey in the UK [32], where women relatives (61% compared to 71% in our study) of older patients (77 years compared to 75 years in our study) were more likely to respond. In addition, 37.5% of UK respondents were spouses compared to 36.8% in our study, and 46.2% were sons or daughters compared to 45.3% in our study.

As we know very little about those who decide not to participate, we can only postulate that bereaved relatives who were not too distressed were comfortable completing the survey, but those who were very distressed found the idea of completing the survey upsetting and did not complete it. As such, risks, needs and scores of PGD could be under-estimated. Koffman et al [34] reported that despite such surveys evoking distressing emotions, many participants found the experience positive and useful, a finding that we will be reporting on in a future publication.

While the survey was population-based, recruitment of bereaved relatives relied on funeral providers wanting to participate and therefore their clients may have different characteristics to the general bereaved population. However, comparative analysis of all variables of respondents from the four funeral providers did not show differences between them. Moreover, the two Australian states, where these four funeral providers are located, are slightly different in their demographic characteristics which provided a sound mix and coverage of the general bereaved population, particularly as respondents came from metropolitan and regional areas of the two states. However, further exploration of different geographical areas and cultural contexts is warranted. Our previous review of bereavement support practices in the United States, Canada, United Kingdom, and Japan demonstrated the same challenges encountered in Australia [28], and therefore this study is likely to have similar implications at the international level.

## Conclusions

This paper provides support for the public health model of bereavement support and is the first of its kind nationally and internationally. The categorization into three groups of need extends the binary approach of either having or not having PGD. This advances the understanding of how three groups have different profiles of need and that most people fit within the low level of risk. Nevertheless, the moderate level of risk (just over a third of bereaved people), which may have been somewhat neglected to date, can now be better understood and interventions can be targeted appropriately.

Appropriate supports and services will ultimately reduce the risk of PGD, through reducing the prevalence of unmet support needs. Currently there is a lack of clear evidence to guide development and allocation of bereavement programs, including programs to develop community capacity. Results from this survey will enable us to fill this gap and determine how the support needs of each of the three groups of bereaved people should be approached. This is of utmost importance for cost-effective and equitable resource allocation, and for understanding the contribution the community at large makes to bereavement support.
